# COVID-19 Identification System Using Transfer Learning Technique With Mobile-NetV2 and Chest X-Ray Images

**DOI:** 10.3389/fpubh.2022.819156

**Published:** 2022-03-03

**Authors:** Mahmoud Ragab, Samah Alshehri, Gamil Abdel Azim, Hibah M. Aldawsari, Adeeb Noor, Jaber Alyami, S. Abdel-khalek

**Affiliations:** ^1^Department of Information Technology, Faculty of Computing and Information Technology, King Abdulaziz University, Jeddah, Saudi Arabia; ^2^Centre of Artificial Intelligence for Precision Medicines, King Abdulaziz University, Jeddah, Saudi Arabia; ^3^Mathematics Department, Faculty of Science, Al-Azhar University, Naser City, Egypt; ^4^Department of Pharmacy Practice, Faculty of Pharmacy, King Abdulaziz University, Jeddah, Saudi Arabia; ^5^Department of Computer Science, Faculty of Computers and Information Canal Suez University, Ismalila, Egypt; ^6^Department of Pharmaceutics, Faculty of Pharmacy, King Abdulaziz University, Jeddah, Saudi Arabia; ^7^Center of Excellence for Drug Research and Pharmaceutical Industries, King Abdulaziz University, Jeddah, Saudi Arabia; ^8^Department of Diagnostic Radiology, Faculty of Applied Medical Sciences, King Abdulaziz University, Jeddah, Saudi Arabia; ^9^Imaging Unit, King Fahd Medical Research Center, King Abdulaziz University, Jeddah, Saudi Arabia; ^10^Department of Mathematics, Faculty of Science, Taif University, Taif, Saudi Arabia; ^11^Department of Mathematics, Faculty of Science, Sohag University, Sohag, Egypt

**Keywords:** machine learning, convolution neural networks, transfer learning, MobileNetV2, COVID-19

## Abstract

Diagnosis is a crucial precautionary step in research studies of the coronavirus disease, which shows indications similar to those of various pneumonia types. The COVID-19 pandemic has caused a significant outbreak in more than 150 nations and has significantly affected the wellness and lives of many individuals globally. Particularly, discovering the patients infected with COVID-19 early and providing them with treatment is an important way of fighting the pandemic. Radiography and radiology could be the fastest techniques for recognizing infected individuals. Artificial intelligence strategies have the potential to overcome this difficulty. Particularly, transfer learning MobileNetV2 is a convolutional neural network architecture that can perform well on mobile devices. In this study, we used MobileNetV2 with transfer learning and augmentation data techniques as a classifier to recognize the coronavirus disease. Two datasets were used: the first consisted of 309 chest X-ray images (102 with COVID-19 and 207 were normal), and the second consisted of 516 chest X-ray images (102 with COVID-19 and 414 were normal). We assessed the model based on its sensitivity rate, specificity rate, confusion matrix, and F1-measure. Additionally, we present a receiver operating characteristic curve. The numerical simulation reveals that the model accuracy is 95.8% and 100% at dropouts of 0.3 and 0.4, respectively. The model was implemented using Keras and Python programming.

## Introduction

The coronavirus disease (COVID-19) has endangered social lives and created disturbing financial costs. Many studies have attempted to find a way to manage its spread and the resulting death. Moreover, many research propositions have been made to evaluate the existence and seriousness of pneumonia triggered by COVID-19 (1–4). Compared to CT, although radiography is easily accessible in hospitals worldwide, X-ray images are considered less delicate for examining patients with COVID-19. Primary analysis is crucial for instant seclusion of the infected individuals. Moreover, it decreases the rate of infection in a healthy population because of the accessibility of sufficient therapy or vaccination for the virus.

Chest radiography is an essential method for identifying pneumonia. This is easy to accomplish with rapid medical identification. Upper body CT has a high level of susceptibility for the identification of COVID-19 (5). The X-ray image reveals aesthetic keys associated with the virus (6, 7). They considered the tremendous rate of suspected individuals and a minimal variety of trained radiologists. These automated identification methods with refined abnormalities help diagnose diseases and raise early medical identification quality with high accuracy.

A therapeutic image in the form of a chest X-ray is crucial for the automatic clinical identification of COVID-19. A clinical approach for identifying COVID-19 based on artificial intelligence (AI) can be as precise as a human, conserve the radiotherapist period, and perform clinical identification less expensively and faster than standard techniques. AI explanations can be influential approaches for dealing with such problems. A machine learning structure was used to predict COVID-19 from a chest X-ray image. In contrast to classic medical image category approaches, which stick to a two-phase treatment (handmade function abstraction plus identification), we used an end-to-end deep determining structure that directly discovers COVID-19 from raw pictures without any pre-processing (8–14). Deep learning (DL)-based variations, specifically convolutional neural networks (CNNs), have been shown to perform better than timeless AI approaches. Recently, various processing system ideas and clinical image evaluation tasks have been utilized in several issues, including categorization, dissection, and face identification (15–19).

Convolutional neural network (CNN) is considered among the finest therapeutic imaging applications (20), particularly for categorization. CNN is better suited for large databases and requires computational assets (storage and memory). In most cases (as in this research), the database is inadequate; therefore, it is insufficient for building and training a CNN. Thus, obtaining the benefit of the CNN's power and transfer learning can reduce the computational cost (21, 22). A proficient CNN on a large and varied image database can perform specific classification tasks (23). Hence, many pre-trained designs such as VGG-Net, (24), Resnet (25), NAS-Net (26), Mobile-Net (27), Inceptions (28), and Xception (29) have won many of the world image classification competitions.

Recently, Bhattachary et al. in (30, 31) presented a survey of most of the DL models in the last five years, used to identify COVID-19 with different data sets. Kermany et al. (32) used the DL framework with transfer learning, Rajaraman et al. (33) used a customized CNN model- VGG16, Wang, et al. (34) proposed AI and DL based Frameworks, Shan et al. (35) presented Human-In-the-Loop Strategy and DL Based Segmentation Network VBNet, Ghoshal and Tucker (36) used Drop weights-based Bayesian Convolutional Network, Apostolopoulos and Mpesiana (37) presented Transfer Learning based on CNN, Esmail et al. (38) used CNN Architecture. Khan et al. (39) presented COVIDX-Net comprising Deep CNN Models. Sahiner et al. (40) proposed Deep CNN Models – ResNet50, InceptionV3, and Inception- ResNetV2.

How can the researchers in poor and developing countries quickly and appropriately contribute to stopping the spread of the virus and thus contributing to the economy's growth? Using AI and machine learning models with transfer learning can contribute to answering this question. This article, which we contribute to, is considered to prevent the spread of the virus.

Because of the absence of a public image database of patients with COVID-19, numerous research studies reporting on the choices for the adaptive discovery of COVID-19 from X-ray images were not easy. This is because they are difficult to train, and many images are required to discover these networks without overfitting. In this work, data augmentation of the training/testing data set was applied.

In this study, we present a COVID-19 X-ray classification technique based on transfer learning with Mobile NetV2, present in the appendix, which is a pre-trained design (without segmentation). This technique addresses the COVID-19 image deficiency issue. As an alternative to training the CNN from the beginning, our method fine tunes the last layer of the design pre-trained variation on Image-NET (presented in Section The Proposed Architecture net and Strategies for Using the Transfer Learning). The above methods assisted in training the versions with conveniently not easily offered images and they attained excellent efficiency.

The MobileNet model proved to be highly efficient in fruit classification with 102 varieties (41, 42) and the classification of systemic sclerosis skin (43). After reviewing the latest modern studies, the latest (30, 31) studies, and to the best of my knowledge, the MobileNet was not used to identify the coronavirus.

The main contribution of this paper is to investigate and evaluate the performance of MobileNetv2 as a lightweight convolutional neural with transfer-learning and augmentation data for the early detection of coronavirus, to propose and adopt a new model structure for MobileNetv2 for binary classification, and to investigate the COVID-Computer-aided diagnostic (CCAD) tool based on the MobilenetV2 developed.

This paper is organized as follows: The methods and materials are described as the general projected framework in Section Materials and Methods. The results and discussions in Section Experimental Results and Discussion describe the database, experimental research studies, and measurement tools. Finally, the conclusions and future work are presented in Section Conclusion and Future Work.

## Materials and Methods

We utilized a CNN to extricate highlights from COVID-19 X-ray images. We took on an extraordinary CNN called a pre-trained show wherever the arrangement is already prepared on the Image-NET database, and it comprised numerous images (creature, plants, …on 1,000 courses). Transfer learning was utilized by exchanging weights obtained and booked right into a pre-trained adaptation system Mobile NetV2.

### Transfer Learning

We had few images in the present task to conduct the training, particularly in the COVID-19 category. Thus, transfer learning is essential. Transfer learning enhances learning in a new job by moving expertise from previous works that have already been reviewed. That is, we used techniques that have been pre-trained on image classification tasks rather than learning a new design. The advantages of transfer learning are as follows:

1. ***Higher begin:*** The first ability (before fine adjustment of the creation) on the resource design is more significant.
2. ***Higher slope:*** The rate of ability enhancement throughout the training of the source design is steeper.
3. ***Higher asymptote:*** The converged skill of the skilled version is much better. When transfer learning is applied to the design, higher precision degrees are quicker.

### Description of Deep CNN

**Figure 1:**
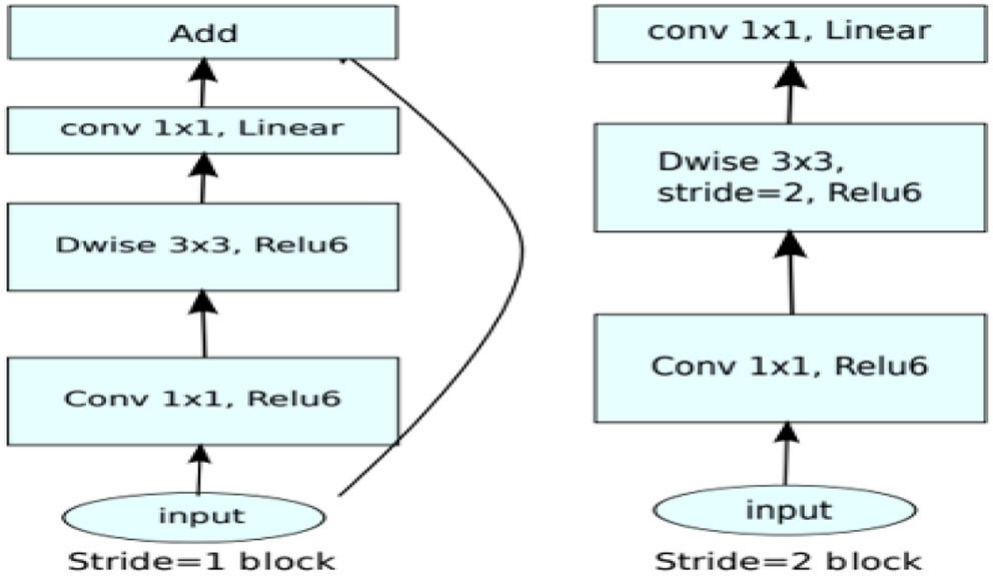
Procedure and flow chart for Mobile-NetV2.

The application system is illustrated in Tables 1, 2, changing the highlights from the M to N channels, with walk s and advancement perspective t. This blockage incorporates a 1 × 1 convolution layer and sometimes the depth-wise convolutional layer. In addition, in requiring the advantage of serial actuation rather than the non-linear actuation included after the pointwise convolutional layer, it fulfills its objective down-sampling by setting basis s within the depth-wise convolutional layer. The entire organized Mobile-NetV2 system (Figure 1) is summarized in Table 2. The two-dimensional (2D) convolution (conv2d) layer is the essential complication, avg-pool is the average pooling, c is the number of the result channels, and n is the copied numbers. CNNs have 19 layers; the center layers are utilized for extricating capacities and the final layers are utilized for categorization. Based on the exchange learning guideline, we utilized Mobile-NetV2 pre-trained by Image-NET as a settled separator.

**Table 1:** Mobile-NetV2 blockage.

**Input**	**Operator**	**Output**
W × H × N	1 × 1 2D convolution (conv2d) layer, ReLU6	W × H × tN
W × H × tN	3 × 3 dwise s = s ReLU6	W/s × H /s × tN
W/s × H/s × tN	Linear 1 × 1 2D convolution (conv2d) layer	W/s × H/s × M

**Table 2:**
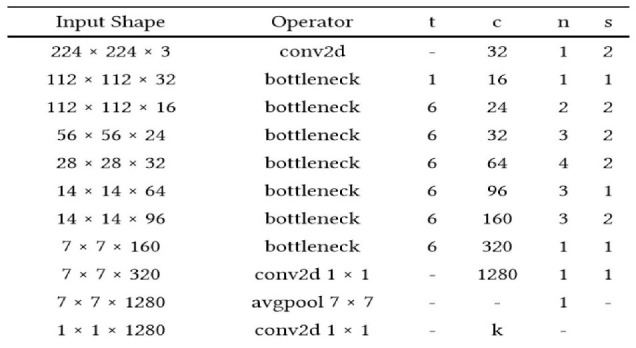
Total network framework of Mobile-NetV2.

**Figure 2:**
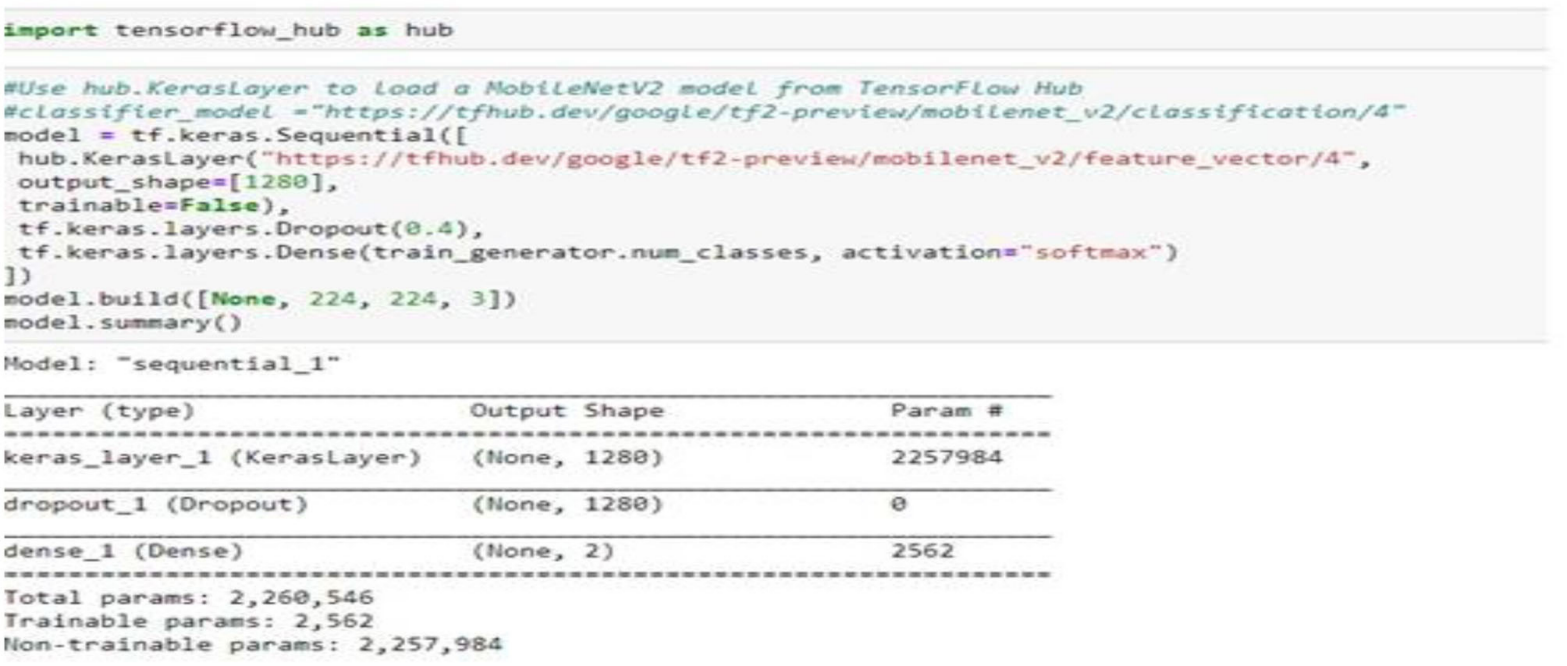
Summary of the proposed model.

The mobile-NetV2 network is a base model without top classification layers, a deal for attribute abstraction. A conv2d layer with a dropout and soft-max categorizer layer is added to the basic design in Figure 2.

The design of the proposed model is summarized in Figure 2; we notice that the total number of coefficients is 2,260,546 for the new model. Only 2,562 coefficients are trainable.

Table 3 shows the differences between the MobileNetV2 model and the other models regarding requirements and efficiency.

**Table 3:** MobileNetV2 and others pre-training models.

**MobileNetV2 and others pre-training models**			
**State-of-the-art Model**	**Parameters**	**Accuracy (Percentage) %**	**Disk Space**
MobileNet	4,253,864	70–89.5	16 MB
MobileNetV2	3,538,984	71–90	14 MB
VGG16	138,357,544	71–90	528 MB
VGG19	143,667,240	71–90	549 MB
ResNet50	25,636,712	74–92	98 MB

## Experimental Results and Discussion

### Database Description

**Figure 3:**
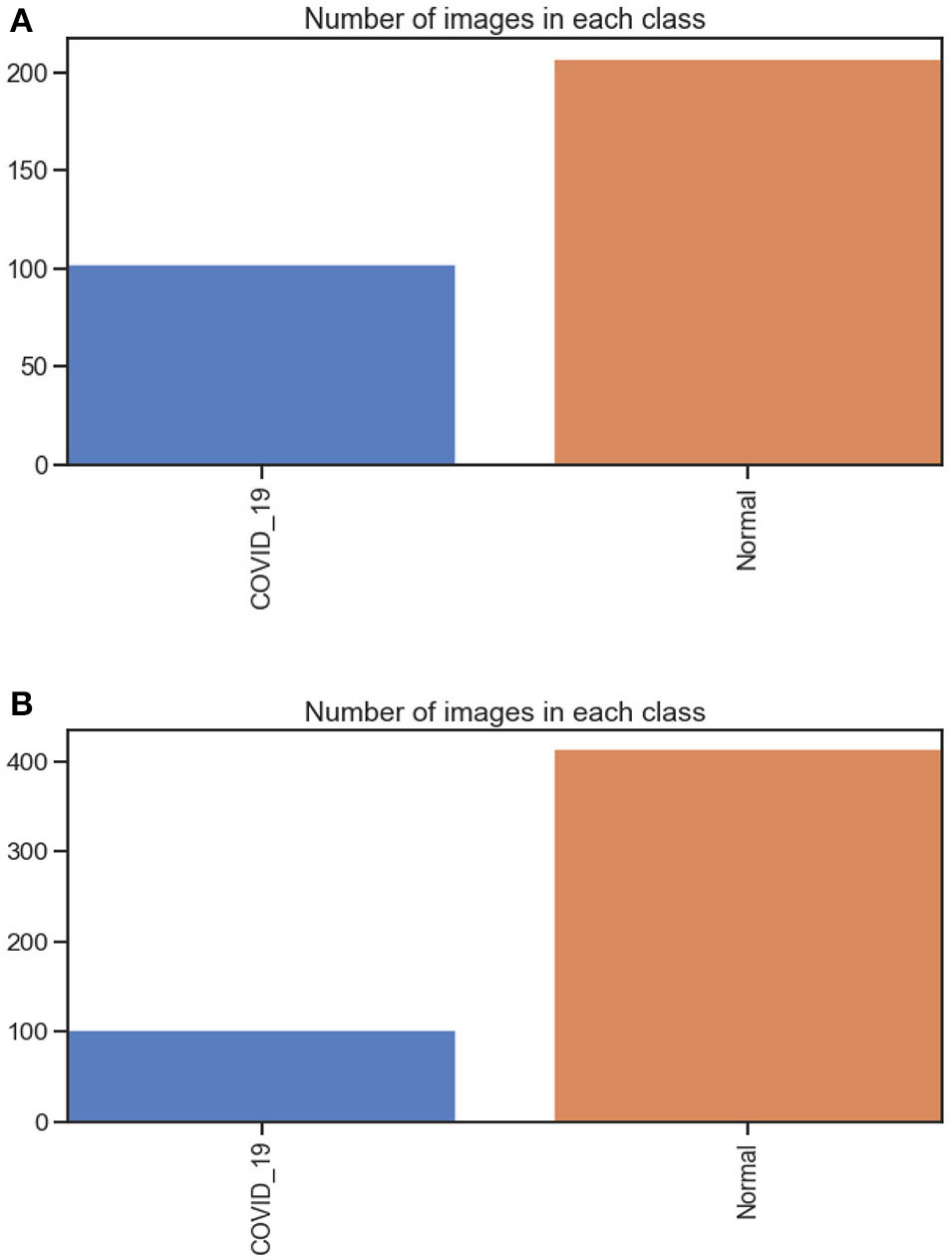
**(A)** number of images in each class dataset A. **(B)** number of images in each class dataset B.

The first dataset includes 309 chest X-ray images (102 were infected with COVID-19 and 207 were normal) see Figure 3. The second dataset includes 516 chest X-rays images (102 were infected with COVID-19 and 414 were normal). All Data sets are organized in two folders, one for training and the second for testing /validation. In each folder of the two (training and testing), two open folders equal to the number of classes, then in each class, the images examples of this class are put. The dataset was obtained from two different open-source databases:

1. GitHub database [https://github (44)].
2. https://kaggle.com/c/rsna-pneumonia-detection-challenge.

They include 8,851 normal samples and 6,012 samples infected with pneumonia, and the chest X-ray image dimension is adjusted to 224 × 224 to satisfy the input conditions of the Mobile-NetV2 model.

Open-source CNN versions are readily available for training from scratch and transfer learning (customizing parts of an extant design for a new job). These versions are frequently tested on the ImageNet dataset of more than 1 million classifieds. The following section presents the proposed structure based on the MobileNetV2.

### The Proposed Architecture Net and Strategies for Using the Transfer Learning

1. **Building the generator of the training data**.

The batches of tensor image data were generated using the class ImageDataGenerator(); also, this class is used as real-time data augmentation with different parameters. With the flow_from_directory() method, which receives two arguments, the directory(from which the images are loaded for creating the batches) and target size (we Adapt the: Target size of the loaded image, which is (256, 256) by default. To (128, 128). In addition, the method receives 3 other arguments (color_mode: RGB, class_mode:' “categorical”, batch_size:32).

2. **Building the validation data generator (is like building the generator of the training data)**.
3. **Loading the MobileNetv2**.

Load the MobileNetv2 from the TensorFlow.Keras.Applications and store in the COVID19Model. Then, remove the last Final conect (FC) layers from the original model. The number of classes is 1,000, and the number of parameters is 4,231,976 trainable parameters in the network. To make this model suitable for working with our data set (Covid19) in two classes: change the size image from (224, 224, 3) to (128, 128, 3). we set in the MobileNet() function argument input_shape = (128, 128), and include_top = False (meaning load the model without FC layers) then make the model return just a vector of length equal to the number of classes. Also, the trainable argument of the loaded model is set to False.

4. **Adding new FC Layers to the modified model**.

We do not need to build a new architecture but use the architecture in the modified model stored in the COVID 19 model. Using the Sequential class in Keras, two layers were added at the top of the modified Mobile Net architecture, which are the: a) Average pooling layer b) FC layer with two neurons using the Dense class. (Note that an FC layer is also called a dense layer, and therefore the class used for building the FC layer is called Dense.) Adapting two layers is better than adapting one layer. Adapting three layers is better than just adapting 1 or 2 layers.

5. **Compiling the new model**.

Use the **compile ()** method with the arguments optimizer, loss and metrics as: optimizer=tf.keras.optimizers.RMSprop (lr=0.0001), loss =“binary_crossentropy” and metrics=[“accuracy”]).

6. **Finally, MobileNet transfer learning over the COVID-19 dataset**.

The **fit–_generator()** method is used. With the argument:

generator: Train generator. steps_per_epoch: set to ceil (number of training samples/batch size). epochs: Number of training epochs. validation_data: Validation generator. validation_steps: calculated as ceil (number of validation samples/batch size.

7. **Finally, save the model with extension.h5**, which is a format for saving structured data.

### Data Augmentation

In machine learning (ML) and deep learning (DL) applications, the availability of large-scale, high-quality datasets plays a major role in the accuracy of the results. Keras ImageDataGenerator class provides a fast and simple method to increase your images. It offers various augmentation strategies like standardization, rotation, shifts, turns, brightness modification, etc. Data augmentation of the training/testing data set was applied to each image. For this, we use used the Keras ImageDataGenerator () class with the flowing set of constructor parameters: Table 4.

**Table 4:** Image data generator () parameters.

**Parameter**	**Value**	**Parameter**	**Value**
*Rescale*	*1/255*	Zca epsilon	1e-06,
Horizontal flip	True	Validation split	0.20
Zoom range	[0.5, 1.0]	Fill mode	nearest
Feature wise center	True	sample wise center	True
Rotation range	90	Width shift range	[−20,20]
Zca whitening	False	Feature wise std normalization,	False
Vertical flip	True	Sample wise std normalization	False,
Channel shift range,	0.0	Height shift range	0.5
Brightness range	[0.5, 1.0],	Shea range,	0.0

### Experimental Environment

To survey the execution of Mobile-NetV2 with exchange learning, TensorFlow 2 (Tensorflow free) was introduced on Windows-10, a 64-bit working framework. Mobile-NetV2 was run using Python (Python free), and it was pre-trained using the Image-NET database. The exploratory framework is as follows: Intel R Center i7-8250U CPU @1.70 GHz, 1900 MHz 7 Core(s). 9 consistent Pr. and the memory was 16 GB. We used the Keras guide (45) and TensorFlow backend (46) in the implementation model.

### Evaluation Metrics

In the classification Models, most researchers used only accuracy as a performance metric. But the accuracy in some cases is not enough because of the imbalance problem in the data. To measure the model's performance and avoid the problem of data imbalance in the classes, we used more than one performance metric, such as the confusion matrix, F1, Precision, Recall, and Area Under Curve (AUC), which is one of the highest used metrics for evaluation. It is used for binary classification problems. The performance of the classification models can be assessed using various metrics, such as classification precision, sensitivity, specificity, F1-score, and accuracy. Sensitivity and specificity can be calculated using the following equations (45–47): Precision: measure the true patterns correctly predicted from the total predicted patterns in a true class. Accuracy: measure the ratio of correct predictions over the total number of examples evaluated, Recall: used to measure the fraction of positive examples that are correctly classified, and F1: a measure that provides the absolute average between accuracy and recall.


$$\begin{array}{c} \text{Accuracy }\left(\text{for each category}\right)=\frac{TN+TP}{TN+TP+FN+FP},\\ \text{Specificity}=\frac{TN}{TN+FP},\text{Sensitivity}=\frac{TP}{FN+TP},\\ \text{Precision}=\frac{TP}{FP+TP},\text{and }F1=\frac{2^{*}TP}{2^{*}TP+FP+FN} \end{array}$$

where true positive (TP) is the correctly categorized number and false positive (FP) is the incorrectly recognized number. False-negative (FN) is the image of a category that is found as an additional class and true negative (TN) is an image that does not belong to any category and is not categorized as any class.

### Evaluation Results

We prepared the experiment with our database utilizing the Adam optimizer and a batch measure of 30. We performed our exploration by selecting a dropout rate (0.4, 0.5). Additionally, we utilized a learning rate rise to 1e-3 for the Adam optimizer and prepared for ten epochs within the pre-training organization; in any case, we select a much lower preparation rate *e*^−5^ for 31 epochs during the fine-adjustment phase.

### Case 1 (Dataset A)

#### Database B

**Figure 4:**
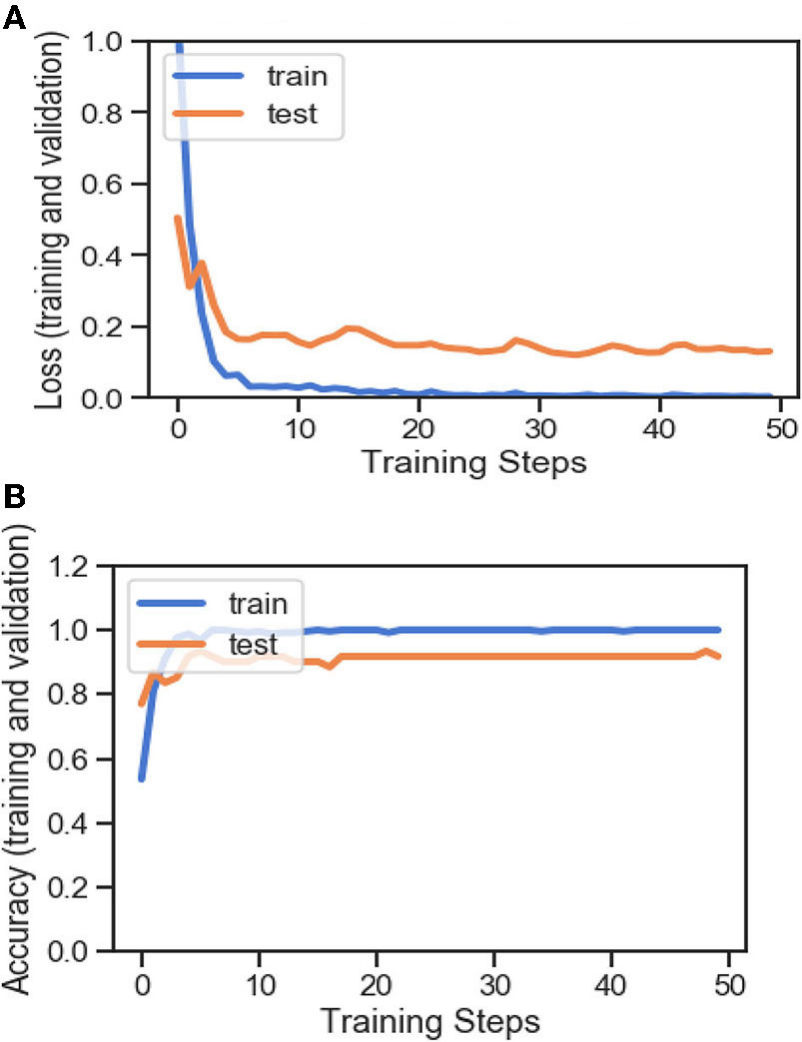
**(A)** Accuracy and loss (training and validation). **(B)** Accuracy and loss (training and validation).

Figures 4A,B, 8A,B, 13A,B, and Table 5 represents the accuracy and loss (training and validation) for data set A, Data set B and Dataset B with only 10 epochs and a dropout of 0.5, respectively.

**Table 5:** Final loss and final accuracy for all data set.

**Data set**	**Final loss**	**Final accuracy**
A	0.09	95.08%
B	0.06	100%
B with only 10 epochs and Dropout 0.5	0.02	99.02%

**Figure 5:**
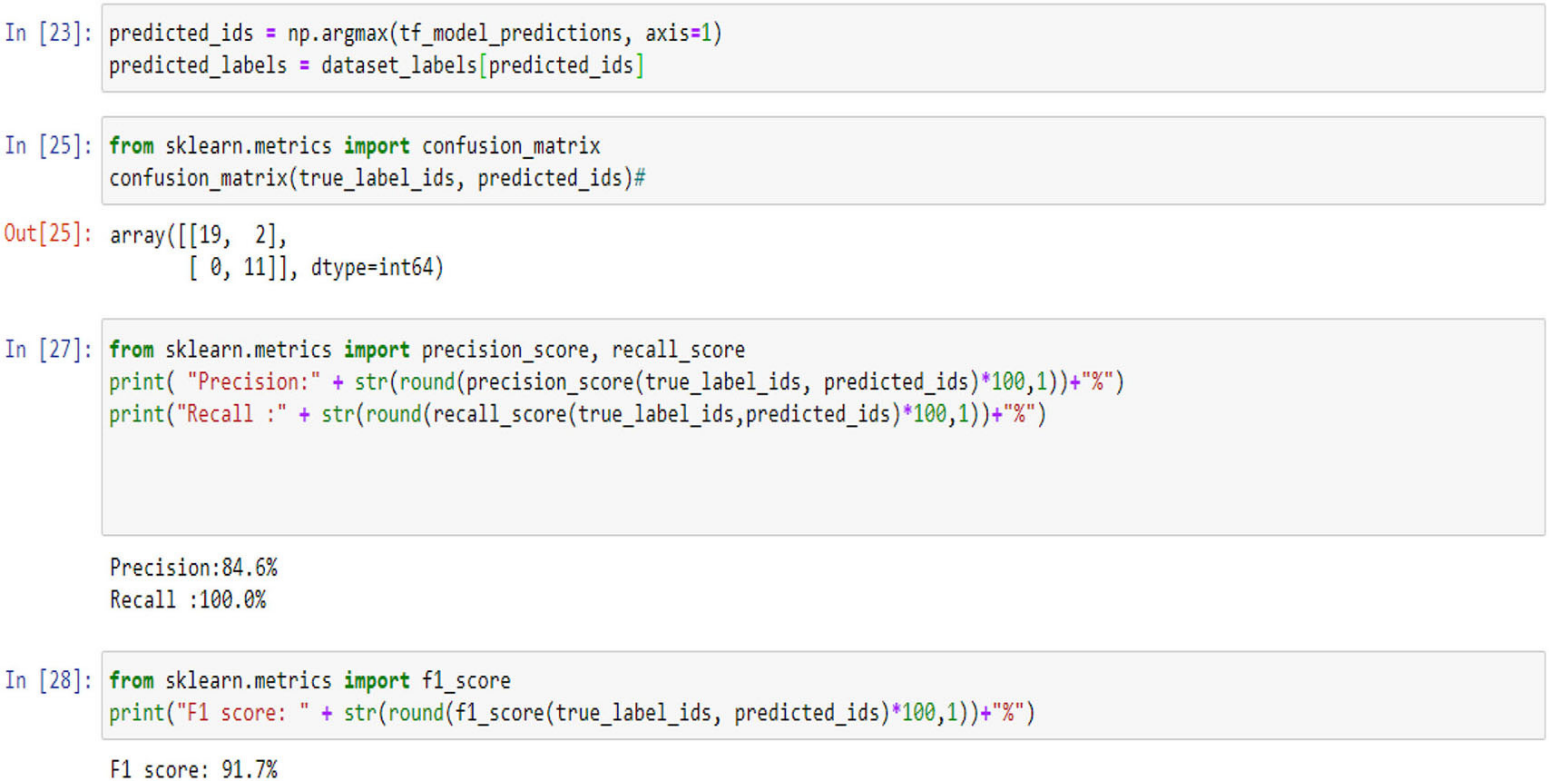
Confusion matrix, precision, recall, and F1 measurements.

Figures 5, 9, 14 and Tables 6, 7 show the Confusion matrix, precision, recall, and F1 measurements for data set A, Data set B, and Dataset B with only 10 epochs and a dropout of 0.5, respectively.

**Table 6:** Precision, recall and F1 score for all data set.

**Data set**	**Precision**	**Recall**	**F1 score**
A	100%	100%	95.0%
B	100%	100%	100%
B with only 10 epochs and Dropout 0.5	100%	100%	100%

**Table 7:** Confusion matrix for all data set.

**Data set**	**Confusion matrix**
A	19 2
	0 11
B	25 0
	0 7
B with only 10 epochs and Dropout of 0.5	25 7
	0 7

**Figure 6:**
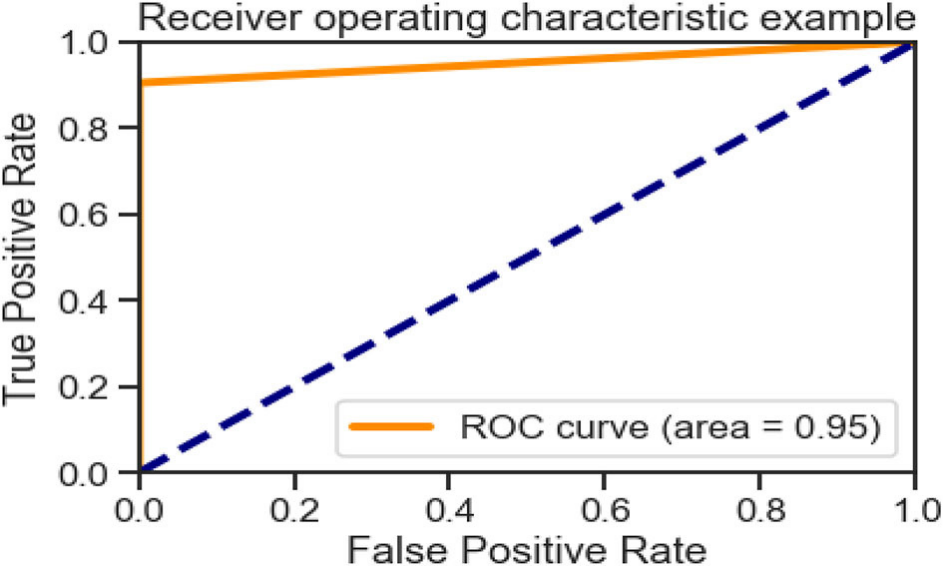
Receiving operating characteristic (ROC) curve.

Figures 6, 10, 15 and Table 8 represents the receiver operating characteristic (ROC) Curve for data set A, Data set B, and Dataset B with only 10 epochs and a dropout of 0.5, respectively (see Figure 12).

**Table 8:** Roc curve area for all data sets.

**Data set**	**Roc curve area**
A	0.95
B	1.0
B with only 10 epochs and Dropout 0.5	1.0

**Figure 7:**
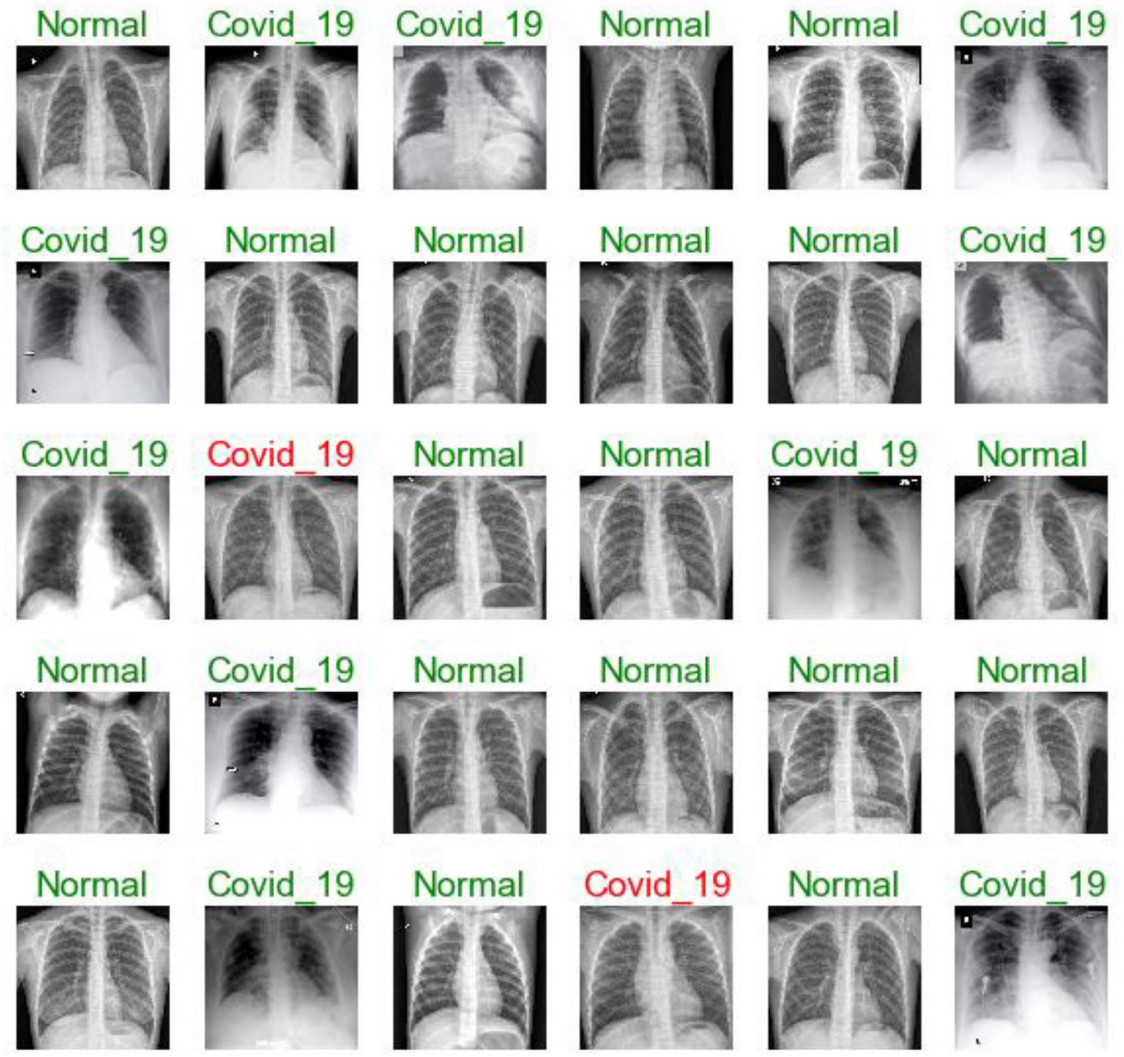
Model predictions (green: correct, red: incorrect).

Figures 7, 11, 16 are present the model predictions results (green: correct, and red: incorrect) for data set A, Data set B, and Dataset B with only 10 epochs and a dropout of 0.5, respectively.

**Figure 8:**
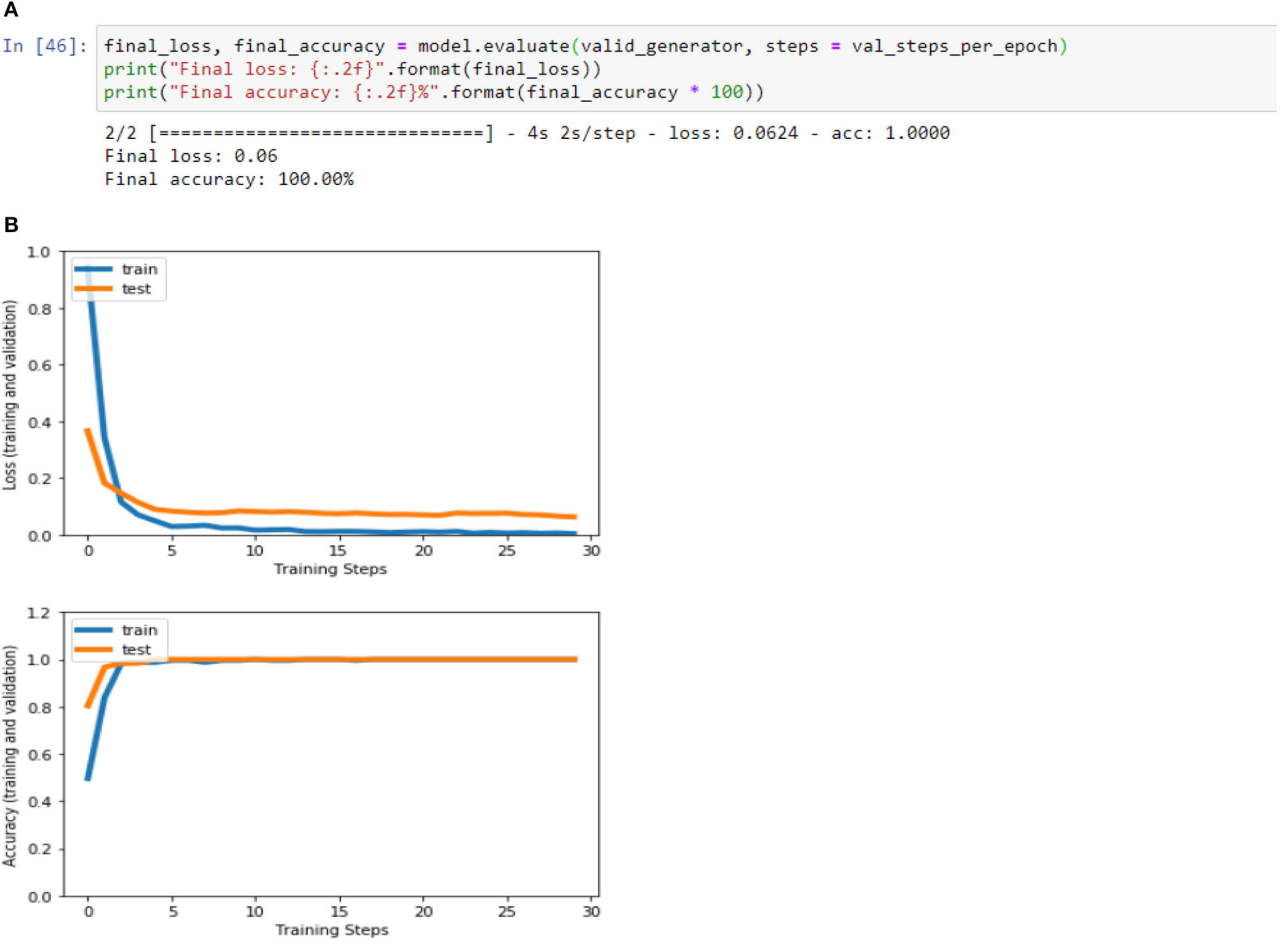
**(A)** Accuracy and loss (training and validation)–Database B. **(B)** Accuracy and loss (training and validation)–Database B.

**Figure 9:**
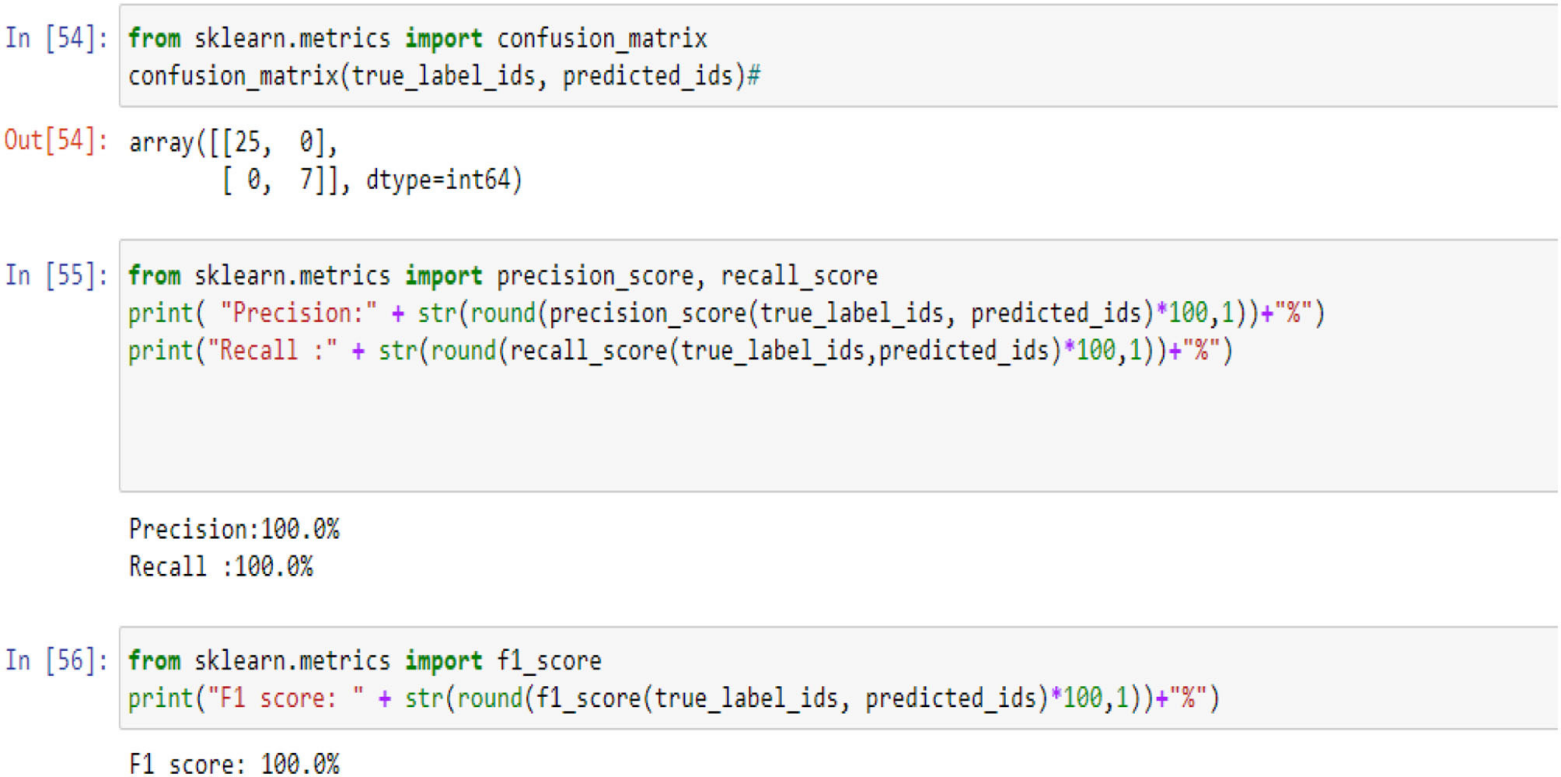
Confusion matrix, precision, recall, and F1 measurements-Case B.

**Figure 10:**
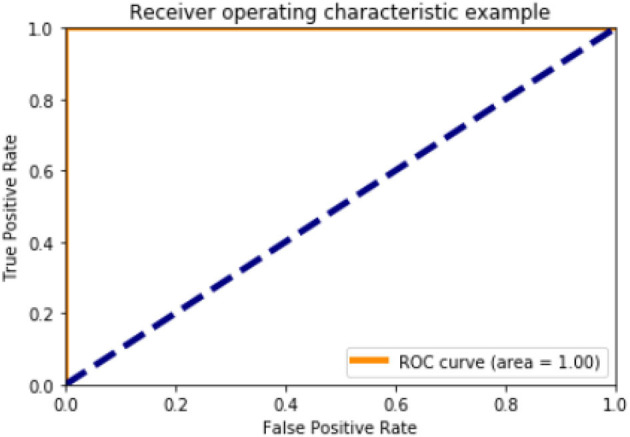
ROC curve (Case B).

**Figure 11:**
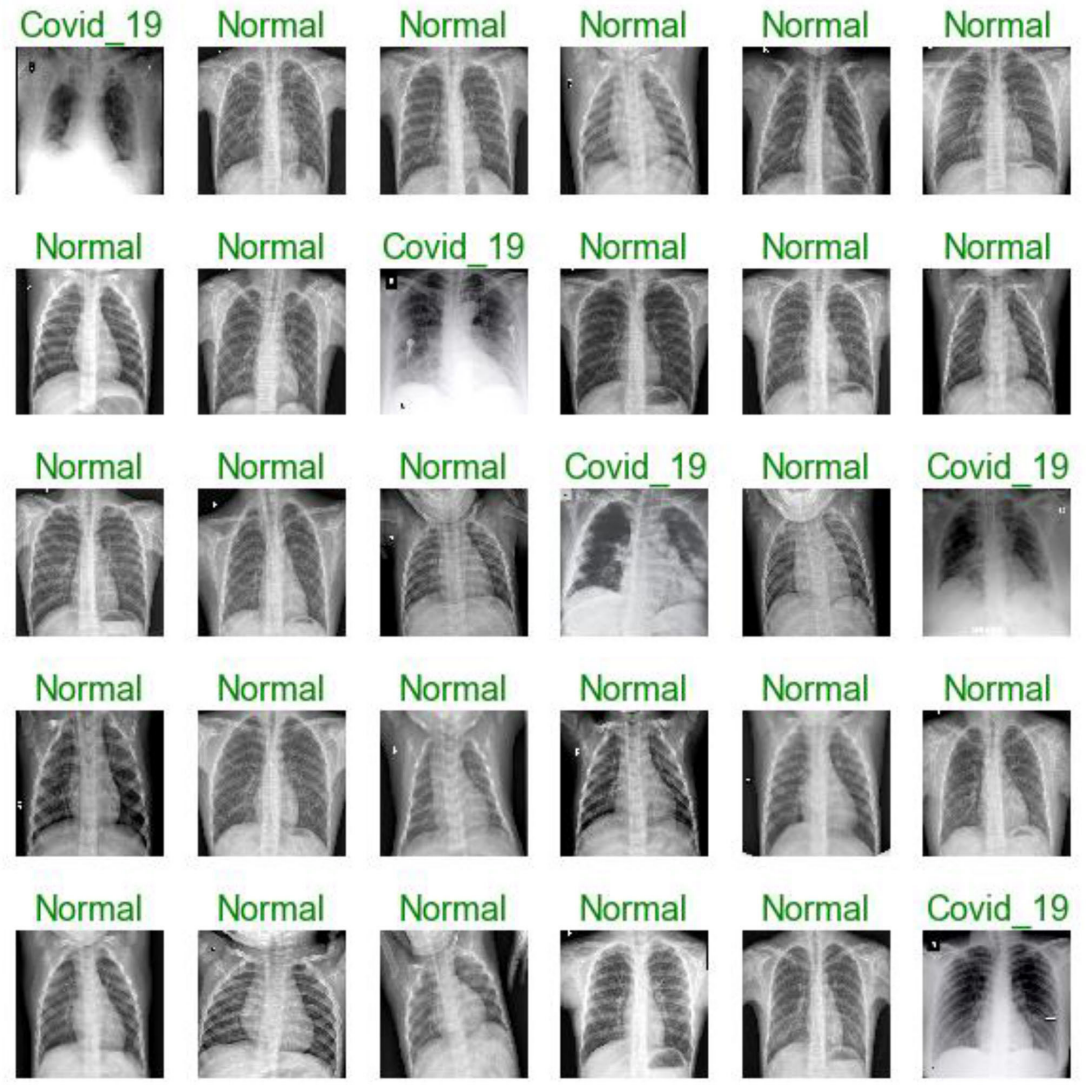
Model predictions (green: correct, red: incorrect).

**Figure 12:**
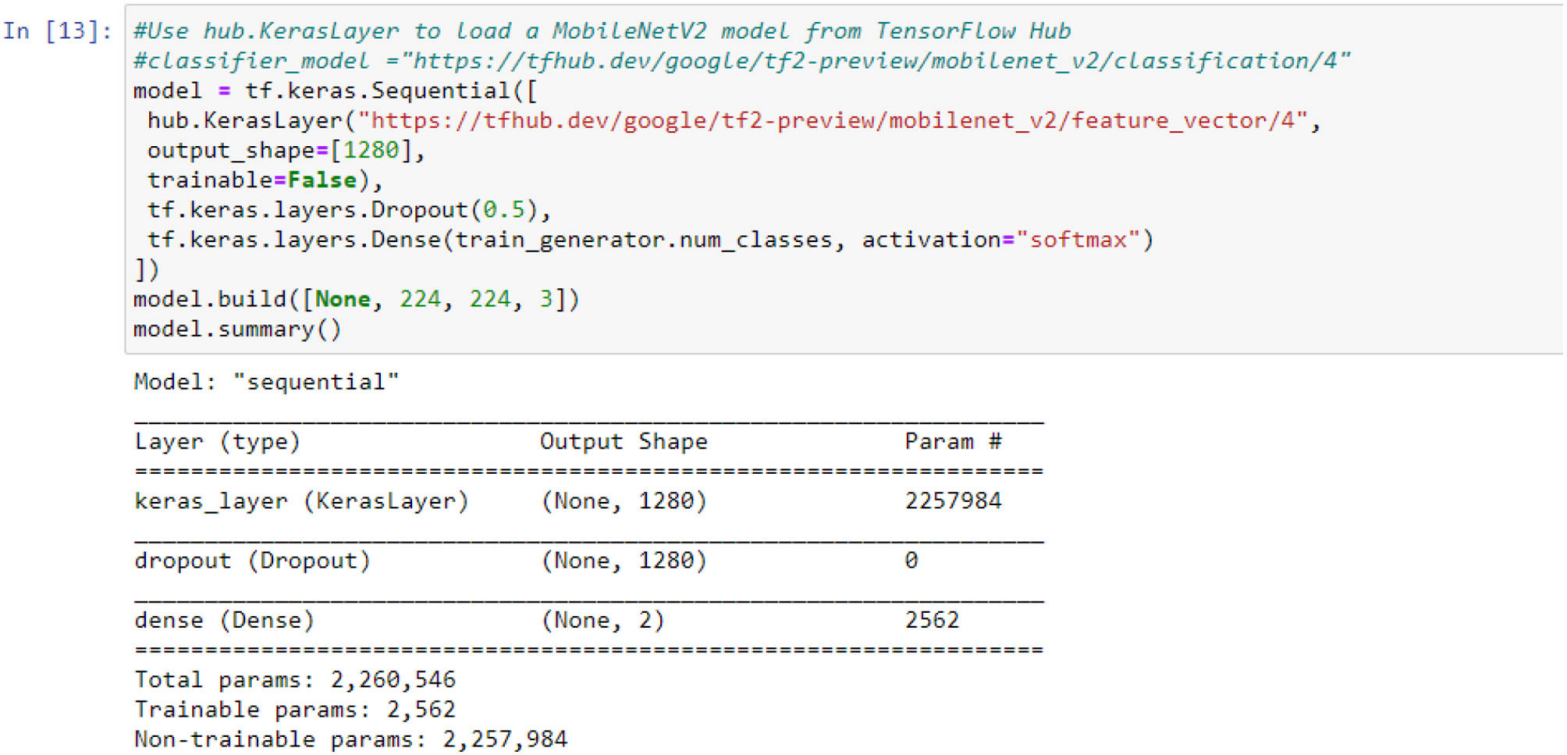
Case B with 10 epochs.

**Figure 13:**
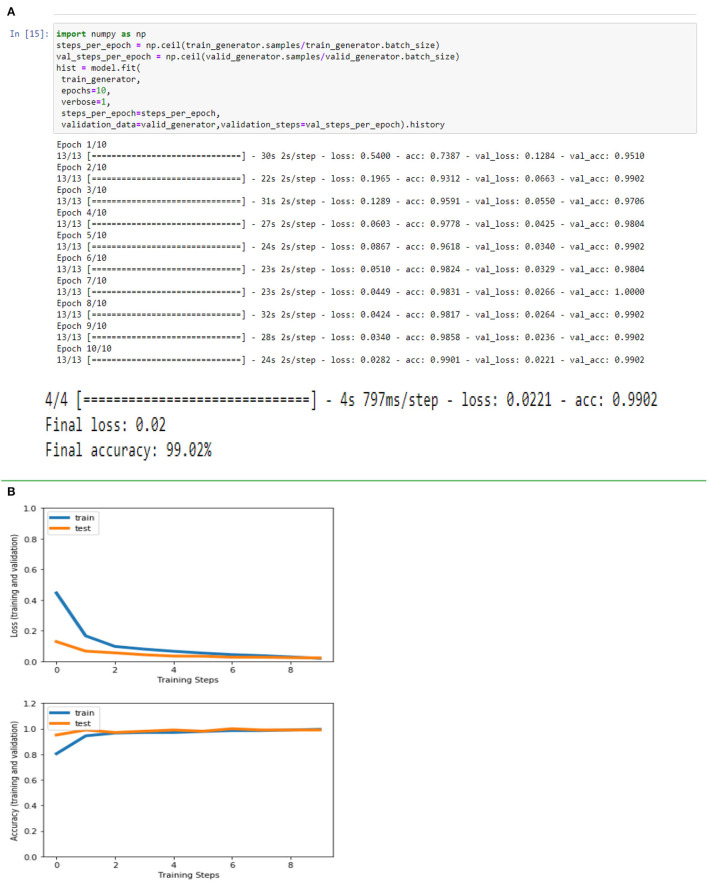
**(A)** Accuracy and loss (training and validation)–Database B with 10 epochs. **(B)** Accuracy and loss (training and validation)–Database B with 10 epochs.

**Figure 14:**
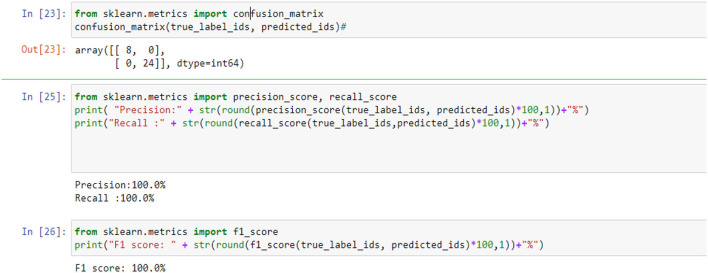
Confusion matrix, precision, recall, and F1 measurements-Case B with 10 epochs.

**Figure 15:**
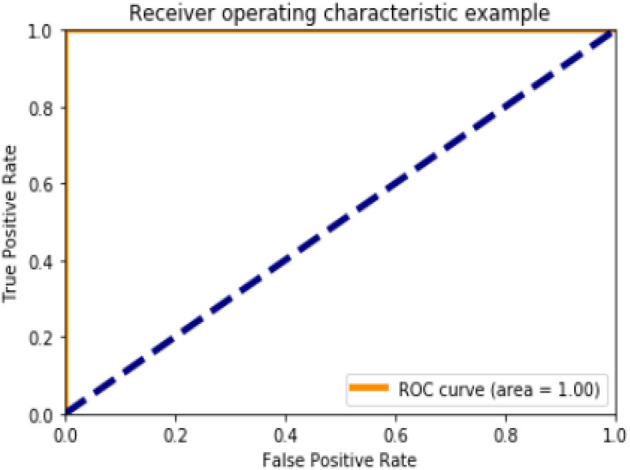
ROC curve (Case B).

**Figure 16:**
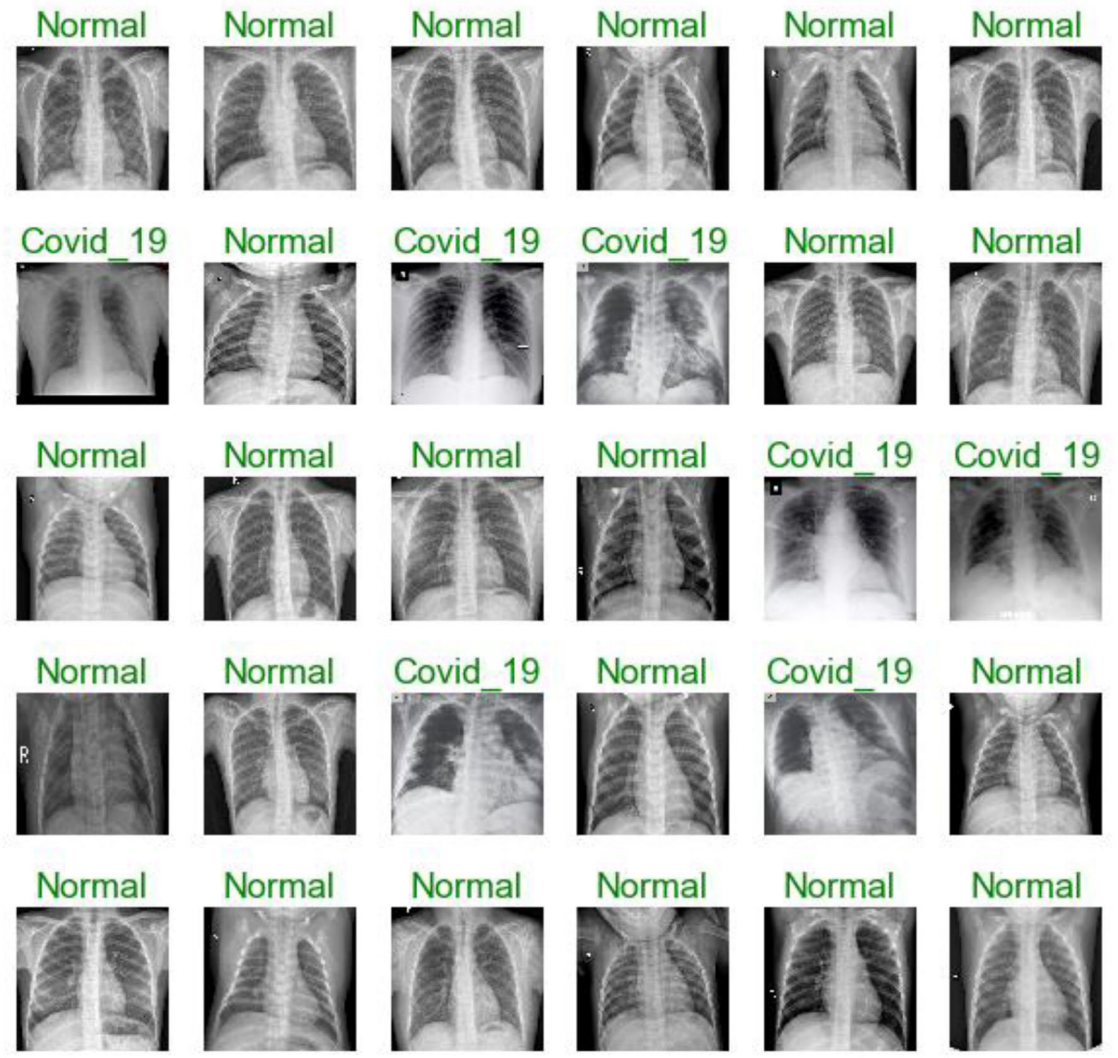
Model predictions (green: correct, red: incorrect).

Based on the results (all figures and tables), it is shown that the proposed classification strategy DL MobileNetV2 might considerably influence the automated detection and extraction of essential features from X-ray images associated with the medical diagnosis of COVID-19.

The preparation of a deep CNN (D-CNN) takes days and hours, and it requires a large database to induce significant precision. Most assignments or information were related. The model coefficients learned can be utilized within the modern demonstration through transfer learning to accelerate and maximize the learning execution of the modern model. We observed from our tests that preparing D-CNNs, particularly for Mobile-NetV2, with a transfer learning strategy could hasten the preparation method.

Moreover, distinct profundity convolutions and modified remaining direct blockages can diminish the number of coefficients. Thus, Mobile-NetV2 can be rapidly sent in versatile and inserted apparatus. Because the database utilized in this study is small, we trained our model utilizing dropout, which is a proficient strategy for minimizing overfitting in neural systems by dodging sophisticated adaptations on preparing information.

### DL Challenge in the Diagnosis of COVID19 Chest Xray

Some limitations of the study research can be overcome in future research, specifically, a more in-depth analysis that requires much more patient data. Establishing models to identify COVID-19 cases from similar viral cases, such as severe acute respiratory syndrome (SARS), and a higher range of common pneumonia is needed. Nonetheless, this work contributes to the possibility of an affordable, fast, and automated diagnosis of the Coronavirus disease.

In the last two years, much DL research on X-rays has been conducted to promote and propose a primary diagnostic tool for COVID-19. These studies have shown good performance results and the best DL algorithm models. These studies have proven that DL algorithms can classify or differentiate between normal and COVID19 chest X-rays. Hence, to keep the study more reliable and to be part of the primary diagnosis tool, perhaps, now is the time to move the study to classify the severity level of COVID-19 where the levels of COVID-19 severity infection are classified into five stages. They are beginning from stage 1 up to stage 5. Stage 1 is defined as the early stage of infection, where these levels classify based on SpO2 level respiratory frequency and difficulty of breathing symptoms. Table 10 shows the severity level and its common symptoms. To certify this future works, deep learning researchers and medical doctors must work together to recognize or build the actual image samples for the algorithm.

## Conclusion and Future Work

This study presents a recognizable verification framework for COVID-19 based on a light neural network, Mobile-NetV2, with a transfer-learning procedure and chest X-ray images. We utilized the pre-trained neural system, Mobile-NetV2, which was prepared using the Image-NET database. The conventional convolution layer was used as the beat layer of a recent study. To reduce overfitting, we connected the recession to the recently included conv2d layer. Mobile-NetV2 demonstrates employment to extricate highlights, and the soft-max categorizer is employed to categorize highlights.

The performance rates indicate that computer-aided diagnostic models based on the convolutional neural MobilenetV2 networks may be employed to diagnose COVID-19. The proposed strategy achieved a distinguishing proof accuracy of 99.9 and 100% in our chest X-ray pictures database, with 309 and 512 images (102 infected with COVID-19, 207 normal; 102 infected with COVID 19, 414 normal, respectively) (see Table 9). The proposed framework can be employed in limited computing and low power devices such as smartphones because Mobile-NetV2 is a lightweight neural arrangement.

**Table 9:** Compare the results obtained using the proposed network with other networks (37).

**Network**		**Accuracy 2-class (%)**	**Sensitivity (%**	**Specificity (%)**	**F1 %**
VGG19 (48)		98.75	92.85	98.75	**-**
MobileNet v2 (28)		97.40	99.10	97.09	**-**
Inception (49)		86.13	12.94	99.70*	**-**
Xception (50)		85.57	0.08	99.99*	**-**
Inception ResNet v2 (49)		84.38	0.01	99.83*	**-**
Proposed Network	A	93.75	100	100	95.0
(Based on MobileNetv2)	B	99.2 and 100	100	100	100

The asterisk * denotes the measurements corresponding to sensitivity and specificity, which obtained not meaningful values due to data imbalance. In our work, Data augmentation is used in the training/testing data set using Keras ImageDataGenerator () class.

**Table 10:** Severity level of COVID-19 (51).

**Severity level**	**Name**	**Description/Individuals who**
1	Asymptomatic	Have no symptoms that are consistent with COVID-19
2	Mild	Have light symptoms
3	Moderate	Show evidence of lower respiratory (SPO2 ≥94%)
4	Severe	Have SPO2 <94%, and other conditions
5	Critical	Have respiratory failure/multiple organ dysfunction

In the future, we plan to promote a hybrid system based on MobileNetV2 as a feature extractor with the Whale Optimization Algorithm (WOA) for efficient feature selection and other classifiers such as sport vector machine or Extra Randomized Tree (ERT) algorithm (52).

## Data Availability Statement

The datasets analysed in this study are available at: GitHub database [https://github (30)] and https://kaggle.com/c/rsna-pneumonia-detection-challenge. Further inquiries can be directed to the corresponding author/s.

## Ethics Statement

Ethics approval and written informed consent were not required for this study in accordance with national guidelines and local legislation.

## Author Contributions

MR supervised the project. GA, HA, and SA designed the model, the computational framework, and analysed the data. AN and MR carried out the implementation. GA and HA performed the calculations. GA and SA wrote the manuscript with input from all authors. All authors provided critical feedback and helped shape the research, analysis and manuscript. All authors contributed to the article and approved the submitted version.

## Funding

This project was supported financially by Institution Fund projects under Grant No. (IFPRC-215-249-2020).

## Conflict of Interest

The authors declare that the research was conducted in the absence of any commercial or financial relationships that could be construed as a potential conflict of interest.

## Publisher's Note

All claims expressed in this article are solely those of the authors and do not necessarily represent those of their affiliated organizations, or those of the publisher, the editors and the reviewers. Any product that may be evaluated in this article, or claim that may be made by its manufacturer, is not guaranteed or endorsed by the publisher.
